# Longitudinal characterization of antimicrobial resistance genes in feces shed from cattle fed different subtherapeutic antibiotics

**DOI:** 10.1186/1471-2180-11-19

**Published:** 2011-01-24

**Authors:** Trevor W Alexander, Jay L Yanke, Tim Reuter, Ed Topp, Ronald R Read, Brent L Selinger, Tim A McAllister

**Affiliations:** 1 Department of Animal Science, University of Vermont, Burlington, Vermont, 05405, USA; 2 Agriculture and Agri-Food Canada Research Centre, Lethbridge, Alberta, T1J 4B1, Canada; 3 Agriculture and Agri-Food Canada Research Centre, London Ontario, N5V 4T3, Canada; 4 Faculty of Medicine, University of Calgary, Calgary, Alberta, T2N 4N1, Canada; 5 Department of Biological Sciences, University of Lethbridge, Lethbridge, Alberta, T1K 3M4, Canada

## Abstract

**Background:**

Environmental transmission of antimicrobial-resistant bacteria and resistance gene determinants originating from livestock is affected by their persistence in agricultural-related matrices. This study investigated the effects of administering subtherapeutic concentrations of antimicrobials to beef cattle on the abundance and persistence of resistance genes within the microbial community of fecal deposits. Cattle (three pens per treatment, 10 steers per pen) were administered chlortetracycline, chlortetracycline plus sulfamethazine, tylosin, or no antimicrobials (control). Model fecal deposits (*n *= 3) were prepared by mixing fresh feces from each pen into a single composite sample. Real-time PCR was used to measure concentrations of *tet*, *sul *and *erm *resistance genes in DNA extracted from composites over 175 days of environmental exposure in the field. The microbial communities were analyzed by quantification and denaturing gradient gel electrophoresis (DGGE) of PCR-amplified *16S-rRNA.*

**Results:**

The concentrations of *16S-rRNA *in feces were similar across treatments and increased by day 56, declining thereafter. DGGE profiles of *16S-rRNA *differed amongst treatments and with time, illustrating temporal shifts in microbial communities. All measured resistance gene determinants were quantifiable in feces after 175 days. Antimicrobial treatment differentially affected the abundance of certain resistance genes but generally not their persistence. In the first 56 days, concentrations of *tet*(B), *tet*(C), *sul1, sul2*, *erm*(A) tended to increase, and decline thereafter, whereas *tet*(M) and *tet*(W) gradually declined over 175 days. At day 7, the concentration of *erm*(X) was greatest in feces from cattle fed tylosin, compared to all other treatments.

**Conclusion:**

The abundance of genes coding for antimicrobial resistance in bovine feces can be affected by inclusion of antibiotics in the feed. Resistance genes can persist in feces from cattle beyond 175 days with concentrations of some genes increasing with time. Management practices that accelerate DNA degradation such as frequent land application or composting of manure may reduce the extent to which bovine feces serves as a reservoir of antimicrobial resistance.

## Background

There is evidence that antimicrobial-resistant (AR) bacteria originating from livestock can be transferred to humans [1,2] thus emphasizing the importance of mitigating their spread into the environment. A critical factor in the dissemination of AR bacteria is persistence in agricultural-related matrices [3]. Most studies on the persistence of AR bacteria in livestock waste have focused on large-scale management systems including stored manure [4] or manure applied to soil [5,6] and have used viable bacteria to describe resistance levels.

While viable indicator bacteria provide useful baseline resistance data, the capacity for bacteria to transfer or acquire antibiotic resistance genes stresses the importance of considering the total level of encoded resistance in a bacterial community [7]. In addition, some bacteria may be intrinsically resistant to a class of antimicrobials, limiting their usefulness in predicting the relevance of resistance expression to dissemination of the trait [8]. DNA-based methods are increasingly being used to monitor the level of resistance genes in environmental samples and have an advantage in that they allow for analysis of community resistance, including bacteria that are un-culturable in the laboratory. Metagenomic studies have been used to examine the prevalence of tetracycline and erythromycin resistance genes in fecal, soil, lagoon and ground water samples in agricultural environments that use antimicrobials [8-11]. However, in some instances these studies lacked detailed information on antimicrobial exposure or the extent to which these determinants persisted over time.

In a previous study, we analyzed AR *Escherichia coli *in artificial fecal deposits originating from animals with a known history of antimicrobial-use [12]. We observed a treatment effect on AR genes encoded by *E. coli *displaying a similar phenotype and also differences in survival of AR genotypes within treatments. In the present study, we sought to extend those findings by determining if differential persistence of AR genes (*tet*, *erm*, *sul*) within the microbial community occurs as a result of the subtherapeutic use of antimicrobials in beef cattle production.

## Results

Antimicrobial resistance genes in fecal deposits from cattle fed subtherapeutic levels of antimicrobial growth promoters were investigated over a 175-day period. The subtherapeutic antimicrobials were selected based on the commonality of use in the industry and included chlortetracycline (44 ppm, A44), chlortetracycline plus sulfamethazine (both at 44 ppm, AS700), tylosin phosphate (11 ppm, T11) or no antibiotic supplementation (control). Resistance genes were quantified by real-time PCR. In addition, differences in bacterial populations, represented by *16S-rRNA*, were analyzed by real-time PCR and DGGE. A detailed description of the complete feedlot experiment has been previously published [12].

### 16S-rRNA genes

**Figure 1:**
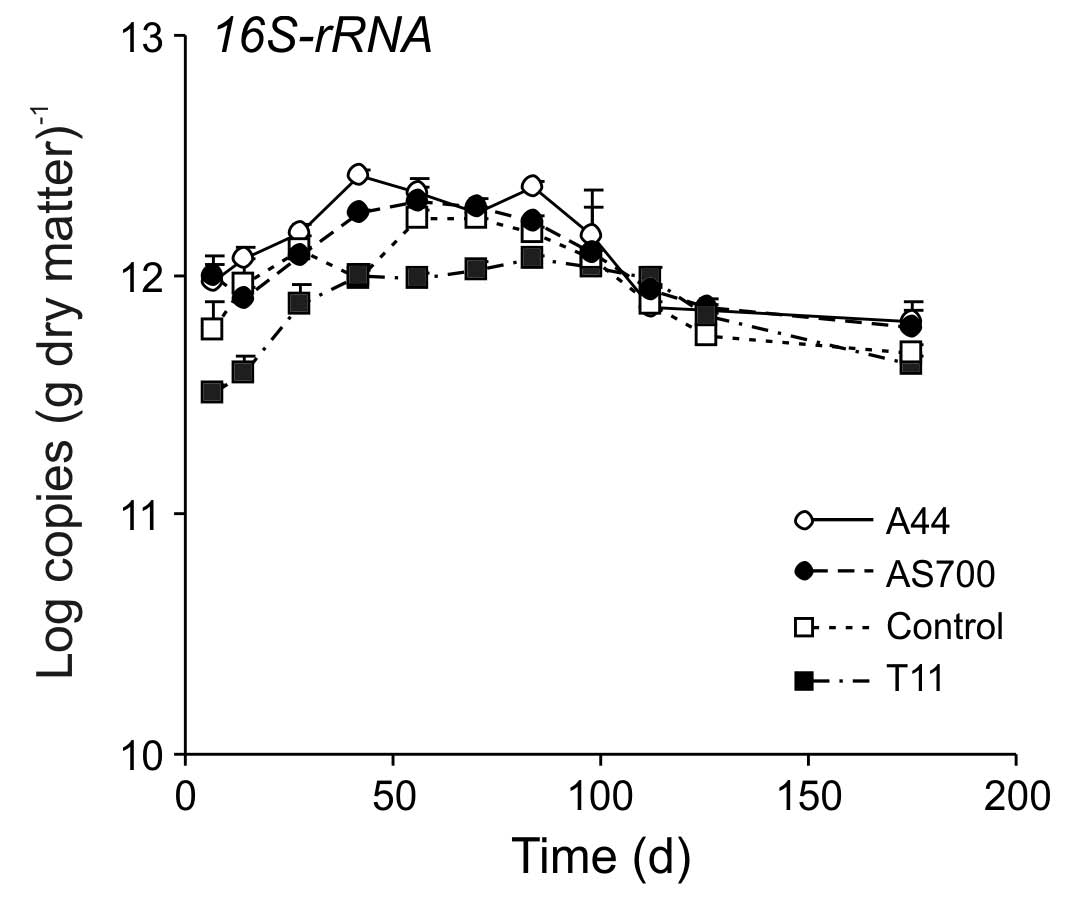
**Quantification of *16S-rRNA *in cattle fecal deposits under field conditions**. The treatments were (N = 3; plus standard error): Control, no antimicrobial agents added to the diets of steers from which fecal deposits originated; A44, chlortetracycline (44 ppm); AS700, chlortetracycline and sulfamethazine (each at 44 ppm); T11, tylosin (11 ppm).

Copies of *16S-rRNA *genes were affected by an interaction between time of fecal pat exposure and treatment (*P *= 0.0001, Figure 1). Generally, the concentration of *16S-rRNA *increased in all treatments by day 56. Concentrations decreased thereafter, but by day 175, were not different from the concentrations on day 7.

### Tetracycline resistance genes

**Figure 2:**
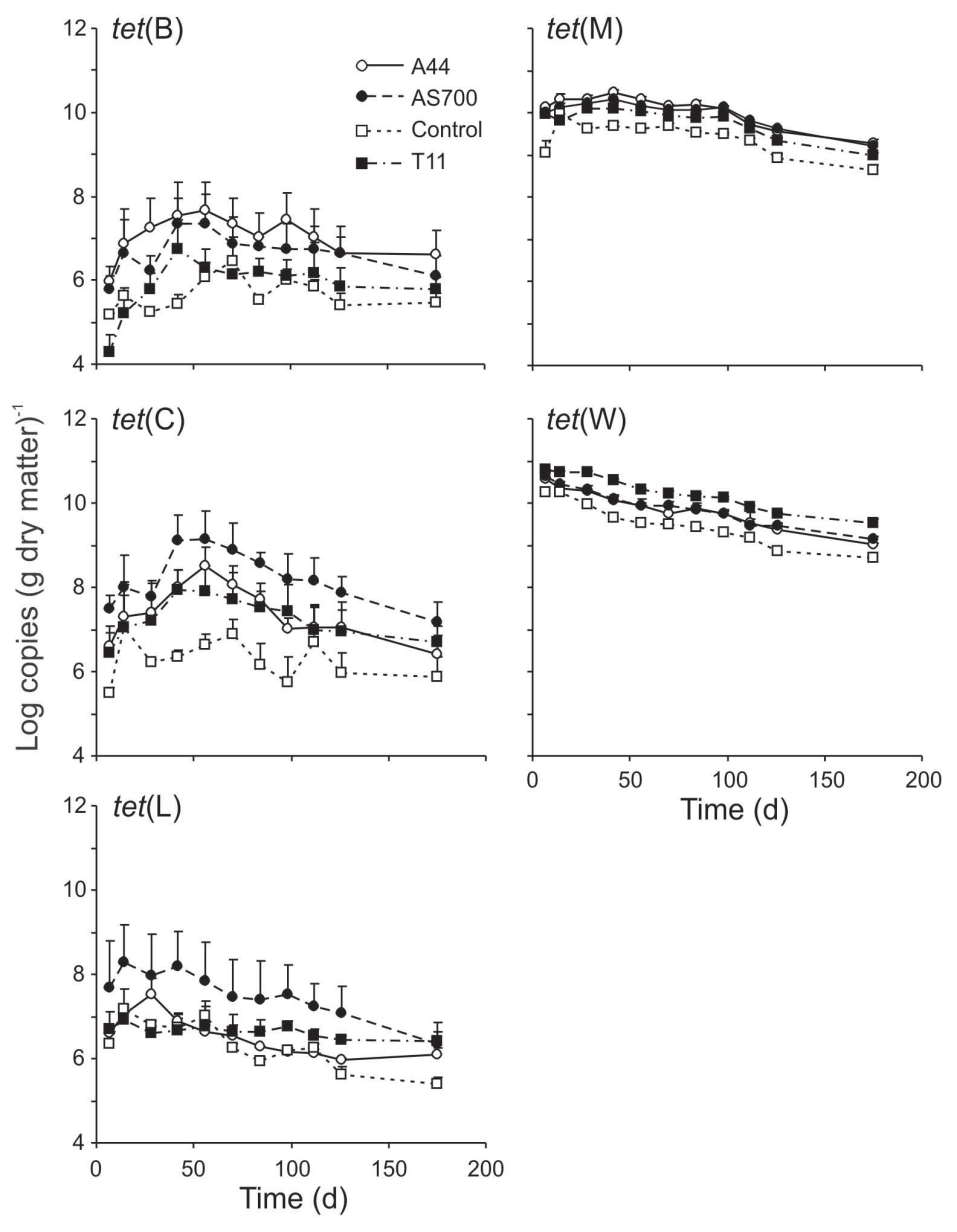
**Persistence of tetracycline resistance genes in cattle fecal deposits under field conditions**. The treatments were (N = 3; plus standard error): Control, no antimicrobial agents added to the diets of steers from which fecal deposits originated; A44, chlortetracycline (44 ppm); AS700, chlortetracycline and sulfamethazine (each at 44 ppm); T11, tylosin (11 ppm).

The concentrations of *tet*(B)*, tet*(C)*, tet*(M) and *tet*(W) in fecal deposits were affected by both treatment and time of exposure (*P *= 0.05, Figure 2). Numbers of copies of *tet*(B) in A44 and AS700 fecal deposits were greater than control and T11 fecal deposits but did not differ between A44 and AS700 treatments. Compared to day 7 levels, the concentration of *tet*(B) increased by day 42 (*P *= 0.01) approximately one order of magnitude and remained greater than day 7 levels up to day 112 (*P *= 0.03), decreasing thereafter. Similarly, the concentration of *tet*(C) increased from initial amounts and was greater between days 42-70 when compared to day 7, but all other time points were not different from day 7. Treatments A44, AS700, and T11 all resulted in greater concentrations of *tet*(C) compared to the control fecal deposits, with AS700 having more copies than all other treatments. The control fecal deposits contained less *tet*(W) compared to the other treatments, but unlike *tet*(C), the T11 fecal deposits had the highest concentration of *tet*(W). After 28 days, the amount of *tet*(W) decreased below the concentration on day 7. Only time (*P *= 0.0001) affected the concentration of *tet*(L) in fecal deposits, which decreased from the initial concentrations on day 7, after 175 days of exposure. An interaction between treatment and time influenced the concentration of *tet*(M). By day 175, copies of *tet*(M) were less in all fecal deposits compared to those on day 7 (*P *= 0.05), with the exception of control samples. There were no differences in *tet*(M) numbers in A44, AS700 or T11 deposits, and all had greater amounts of *tet*(M) on day 7 as compared to control deposits. However, by day 112, the fecal deposits had similar *tet*(M) concentrations. Although not analyzed statistically, the concentrations of *tet*(M) and *tet*(W) were greater than other tetracycline resistance determinants.

### Sulfonamide resistance genes

**Figure 3:**
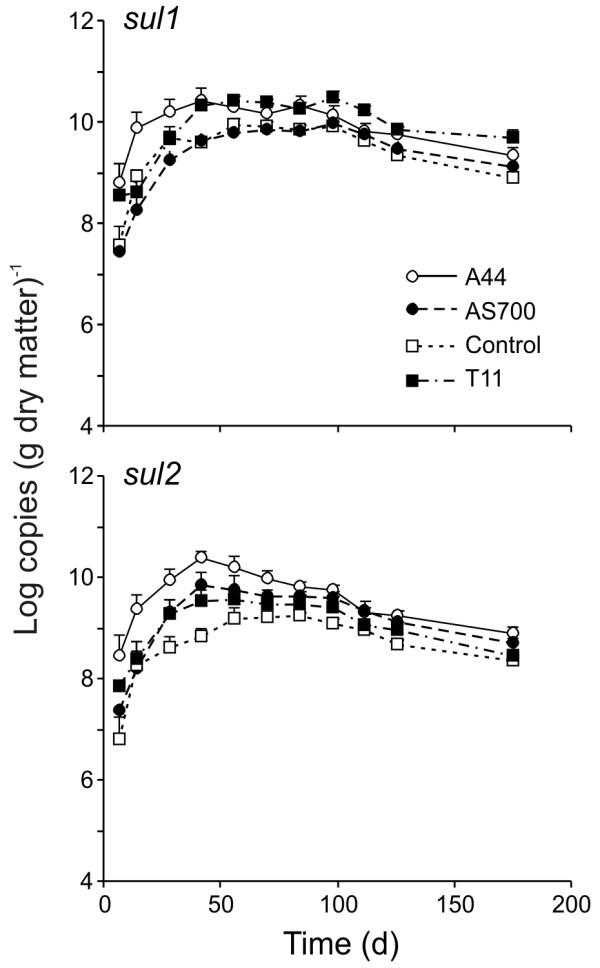
**Persistence of sulfonamide resistance genes in cattle fecal deposits under field conditions**. The treatments were (N = 3; plus standard error): Control, no antimicrobial agents added to the diets of steers from which fecal deposits originated; A44, chlortetracycline (44 ppm); AS700, chlortetracycline and sulfamethazine (each at 44 ppm); T11, tylosin (11 ppm).

An interaction between treatment and time affected the resistance determinant *sul1 *in fecal deposits (P = 0.0001, Figure 3). Concentrations increased 1-2 order of magnitude Log_10 _copies (g DM)^-1 ^within the first 56 days of the experiment, across all treatments, and remained greater on day 175 than the starting concentrations on day 7 (*P *= 0.05). The exception was the A44 treatment, which had similar levels of *sul1 *on day 7 and day 175. On day 14, *sul1 *levels were greater in A44 fecal deposits compared to all other treatments but from day 28 onwards, there were no differences between treatments. Quantified *sul2 *determinants displayed a similar trend to *sul1*. There was an interaction between treatment and time (*P *= 0.001) and *sul2 *concentrations in fecal deposits from all treatments increased in the first 42 days. Levels of *sul2 *in AS700 and control fecal deposits on day 175 were greater than day 7 whereas in treatment A44 and T11 deposits, the concentration of *sul2 *decreased by day 175 and were not different than day 7. Solely the A44 treatment showed greater numbers of *sul2*, in comparison to the control, and only from days 0-42.

### Erythromycin resistance genes

**Figure 4:**
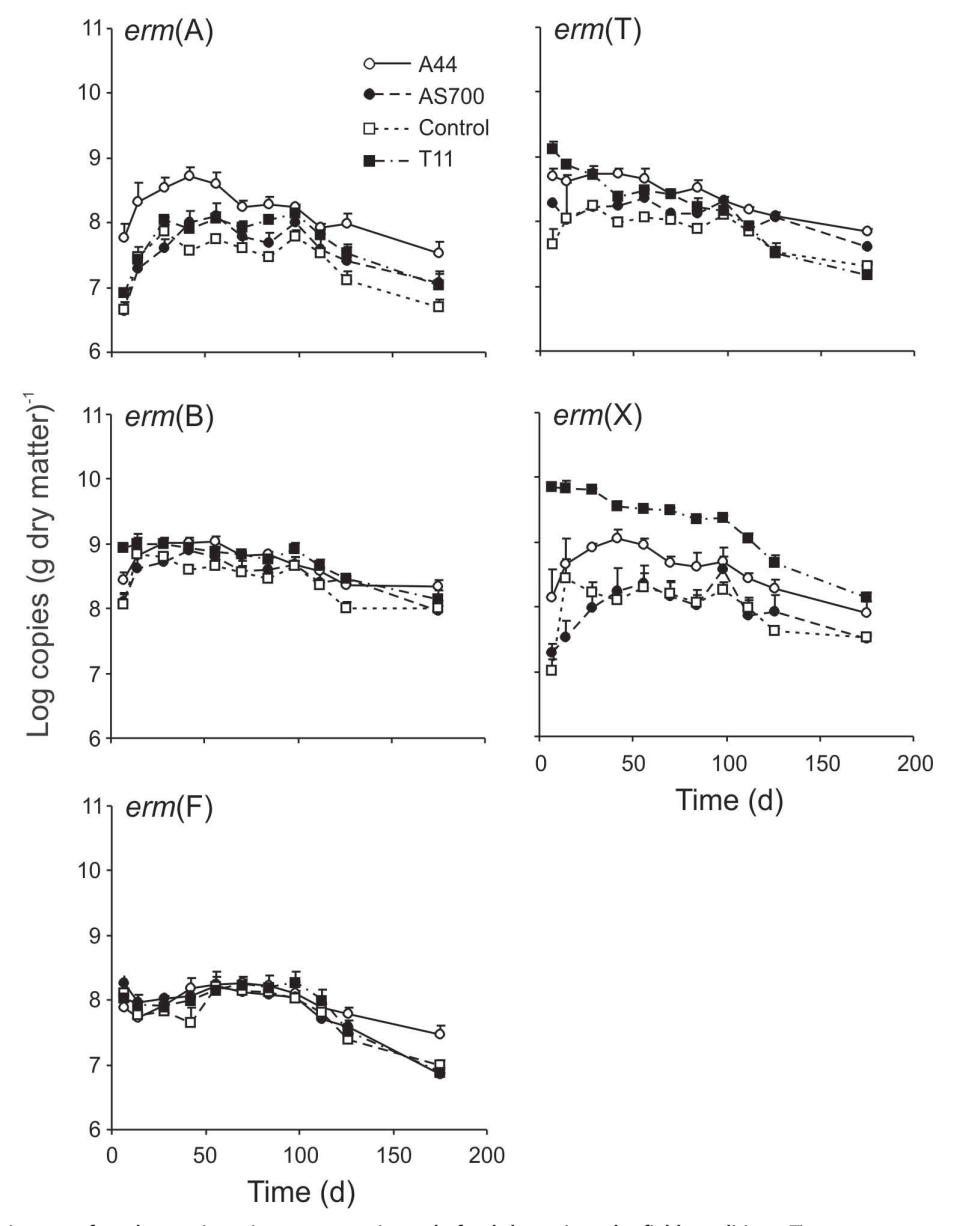
**Persistence of erythromycin resistance genes in cattle fecal depostis under field conditions**. The treatments were (N = 3; plus standard error): Control, no antimicrobial agents added to the diets of steers from which fecal deposits originated; A44, chlortetracycline (44 ppm); AS700, chlortetracycline and sulfamethazine (each at 44 ppm); T11, tylosin (11 ppm).

Every *erm *gene quantified was affected by an interaction between treatment and time of exposure (*P *= 0.05, Figure 4). For *erm*(A), the concentrations increased in all treatments and remained greater than the day 7 values up to day 84. By day 175, the concentrations were not different from those on day 7. With the exceptions of days 98 and 112, the *erm*(A) in A44 fecal deposits were always greater than control samples and were also greater than the concentrations in AS700 and A44 for the first 42 days. Similar to *erm*(A), the concentrations of *erm*(B) and *erm*(X) in control, A44, and AS700 deposits initially increased up to days 42-56 and then decreased to levels comparable to day 7. For both determinants, the concentrations decreased in T11 fecal deposits. Quantified *erm*(B) and *erm*(X) were greater in T11 deposits compared to all other treatments on day 7 and days 7-98, respectively. In both A44 and T11 fecal deposits, the concentration of *erm*(T) were greater than control deposits on day 7 only. Amounts of *erm*(T) decreased by day 175. This was similar to *erm*(F), which decreased by day 175 in all deposits except for A44 samples.

### Denaturing gradient gel electrophoresis (DGGE)

**Figure 5:**
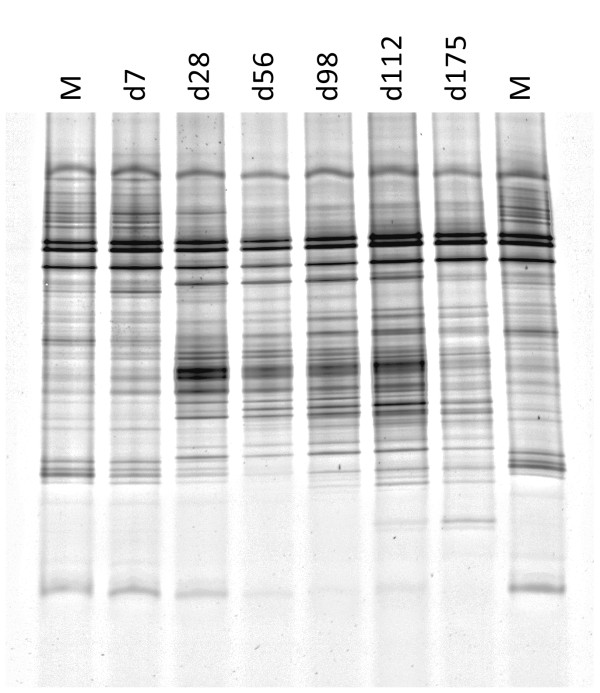
**Representative DGGE profiles generated from PCR-amplified *16S-rRNA *in fecal deposits from the control group of cattle**. DNA from replicate fecal deposits (N = 3) were pooled for analysis. The time points were days (d) 7, 28, 56, 98, 112, and 175. M, marker used to normalize gels consisted of pooled DNA from all treatments on days 7 and 175.

**Figure 6:**
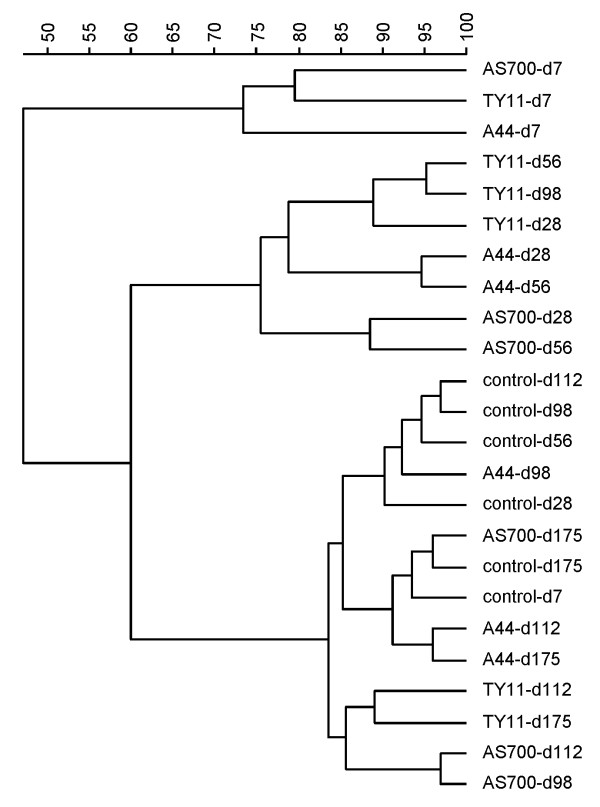
**Similarity of DGGE profiles generated from PCR-amplified *16S-rRNA *in cattle fecal deposits under field conditions**. DNA from replicate fecal deposits (N = 3) were pooled for analysis. The time points were days (d) 7, 28, 56, 98, 112, and 175. The treatments were: Control, no antimicrobial agents added to the diets of steers from which fecal deposits originated; A44, chlortetracycline (44 ppm); AS700, chlortetracycline and sulfamethazine (each at 44 ppm); T11, tylosin (11 ppm).

Representative results showing DGGE profiles from control samples are shown in Figure 5. When comparing all treatments, the DGGE profiles grouped into three main clusters (Figure 6). One cluster only consisted of day 7 DGGE profiles from A44, AS700, and T11 treatments and was least related to other DGGE profiles (42% average similarity). A second cluster also contained solely profiles from treatments A44, AS700, and T11 on days 28, 56, and 98 (average within group similarity 76%). Profiles of the third cluster were most related (average within group similarity 84%) and contained DGGE profiles from all fecal samples.

### Correlations between gene copy concentrations

Numerous correlations between the analyzed genes were significant (*P *< 0.05, Tables 1, 2, 3, and 4). Several were seen across all treatments and included the positive associations between *erm*(T) and *tet*(M) (r = 0.69 to 0.87), *sul1 *and *sul2 *(r = 0.80 to 0.95), and *tet*(M) and *tet*(W) (r = 0.56 to 0.79). From all treatments, the determinants *tet*(B), *tet*(C), and *tet*(L) were not associated. Other than the correlation between *sul1 *and *sul2*, the strongest correlations observed were between genes *erm*(B), *erm*(T), and *erm*(X) ( r = 0.85 to 0.94) and the genes *tet*(W) and *erm*(T) (r = 0.92) within the T11 treatment.

**Table 1:** Pearson correlation coefficient between antimicrobial resistance or *16S-rRNA *genes in fecal deposits from cattle fed no (control) subtherapeutic antimicrobial agents^a^.

	*tet*(C)	*tet*(L)	*tet*(M)	*tet*(W)	*sul1*	*sul2*	*erm*(A)	*erm*(B)	*erm*(F)	*erm*(T)	*erm*(X)	*16S-rRNA*
*tet*(B)	0.29	0.08	0.22	-0.10	0.40*	0.47*	0.34	0.26	0.30	0.24	0.45*	0.41*
*tet*(C)		0.13	0.44*	0.03	0.12	0.29	0.17	0.44*	0.04	0.52*	0.43*	0.23
*tet*(L)			0.49*	0.62*	0.05	-0.06	0.46*	0.65*	0.34	0.24	0.31	0.29
*tet*(M)				0.56*	0.39*	0.36*	0.70*	0.66*	0.52*	0.74*	0.64*	0.76*
*tet*(W)					-0.32	-0.42*	0.18	0.37*	0.53*	0.37*	0.11	0.31
*sul1*						0.92*	0.78*	0.36*	0.20	0.36*	0.61*	0.64*
*sul2*							0.71*	0.41*	0.20	0.45*	0.72*	0.59*
*erm*(A)								0.72*	0.46*	0.63*	0.78*	0.74*
*erm*(B)									0.39*	0.67*	0.77*	0.50*
*erm*(F)										0.54*	0.32	0.70*
*erm*(T)											0.70*	0.66*
*erm*(X)												0.59*

**a**. Analysis was performed across time points, described in the Materials and Methods. Values were log-transformed before correlations analysis. *, *P *≤ 0.05.

**Table 2:** Pearson correlation coefficient between antimicrobial resistance or *16S-rRNA *genes in fecal deposits from cattle fed subtherapeutic levels of chlortetracycline (A44)^a^.

	*tet*(C)	*tet*(L)	*tet*(M)	*tet*(W)	*sul1*	*sul2*	*erm*(A)	*erm*(B)	*erm*(F)	*erm*(T)	*erm*(X)	*16S-rRNA*
*tet*(B)	-0.23	0.08	0.27	-0.14	0.39*	0.36*	0.29	0.32	0.43*	0.10	0.06	0.45*
*tet*(C)		0.19	0.48*	0.24	0.42*	0.56*	0.48*	0.57*	0.01	0.37*	0.70*	0.41*
*tet*(L)			0.56*	0.60*	0.02	0.14	0.31	0.59*	-0.04	0.53*	0.41*	0.30
*tet*(M)				0.79*	0.43*	0.55*	0.71*	0.80*	0.43*	0.87*	0.69*	0.75*
*tet*(W)					-0.05	0.06	0.35*	0.47*	0.17	0.82*	0.39*	0.36*
*sul1*						0.94*	0.82*	0.64*	0.48*	0.37*	0.73*	0.67*
*sul2*							0.85*	0.76*	0.49*	0.44*	0.82*	0.76*
*erm*(A)								0.80*	0.51*	0.72*	0.84*	0.69*
*erm*(B)									0.44*	0.71*	0.81*	0.80*
*erm*(F)										0.44*	0.27	0.68*
*erm*(T)											0.64*	0.61*
*erm*(X)												0.61*

**a**. Analysis was performed across time points, described in the Materials and Methods. Values were log-transformed before correlations analysis. *, *P *≤ 0.05.

**Table 3:** Pearson correlation coefficient between antimicrobial resistance or *16S-rRNA *genes in fecal deposits from cattle fed subtherapeutic levels of a mixture of chlortetracycline and sulfamethazine (AS700)^a^.

	*tet*(C)	*tet*(L)	*tet*(M)	*tet*(W)	*sul1*	*sul2*	*erm*(A)	*erm*(B)	*erm*(F)	*erm*(T)	*erm*(X)	*16S-rRNA*
*tet*(B)	0.23	-0.05	0.16	-0.23	0.40*	0.46*	0.18	-0.08	0.01	0.30	-0.07	0.18
*tet*(C)		-0.31	0.38*	0.24	0.55*	0.65*	0.77*	0.49*	0.40*	0.09	0.69*	0.63*
*tet*(L)			0.42*	0.20	-0.26	-0.28	-0.19	0.41*	0.34	0.46*	-0.18	0.05
*tet*(M)				0.68*	0.08	0.23	0.45*	0.67*	0.87*	0.73*	0.36*	0.70*
*tet*(W)					-0.48*	-0.29	0.02	0.36*	0.73*	0.47*	0.07	0.35*
*sul1*						0.95*	0.80*	0.34	-0.04	-0.03	0.66*	0.46*
*sul2*							0.86*	0.42*	0.09	0.08	0.69*	0.58*
*erm*(A)								0.68*	0.34*	0.17	0.87*	0.70*
*erm*(B)									0.58*	0.46*	0.67*	0.58*
*erm*(F)										0.77*	0.34	0.72*
*erm*(T)											0.15	0.52*
*erm*(X)												0.60*

**a**. Analysis was performed across time points, described in the Materials and Methods. Values were log-transformed before correlations analysis. *, *P *≤ 0.05.

**Table 4:** Pearson correlation coefficient between antimicrobial resistance or *16S-rRNA *genes in fecal deposits from cattle fed subtherapeutic levels of tylosin (T11)^a^.

	*tet*(C)	*tet*(L)	*tet*(M)	*tet*(W)	*sul1*	*sul2*	*erm*(A)	*erm*(B)	*erm*(F)	*erm*(T)	*erm*(X)	*16S-rRNA*
*tet*(B)	0.02	0.24	-0.08	-0.24	0.64*	0.62*	0.57*	0.10	0.09	-0.25	-0.12	0.68*
*tet*(C)		-0.29	0.61*	-0.01	0.46*	0.64*	0.37*	0.18	0.34	0.02	0.14	0.42*
*tet*(L)			-0.02	0.25	0.09	-0.08	0.19	0.30	0.31	0.31	0.30	0.01
*tet*(M)				0.67	0.14	0.43*	0.47*	0.79*	0.72*	0.69*	0.81*	0.32
*tet*(W)					-0.43*	-0.15	0.05	0.80*	0.47*	0.92*	0.91*	-0.19
*sul1*						0.80*	0.69*	-0.04	0.27	-0.39*	-0.19	0.82*
*sul2*							0.84*	0.28	0.46*	-0.09	0.07	0.88*
*erm*(A)								0.44*	0.61*	0.12	0.30	0.85*
*erm*(B)									0.73*	0.85*	0.89*	0.24
*erm*(F)										0.65*	0.72*	0.48*
*erm*(T)											0.94*	-0.13
*erm*(X)												0.03

**a**. Analysis was performed across time points, described in the Materials and Methods. Values were log-transformed before correlations analysis. *, *P *≤ 0.05.

## Discussion

This study investigated the prevalence and persistence of antimicrobial resistance genes sampled from cattle feces under ambient field conditions. The analyzed fecal samples were representative of feedlot practices in which waste can accumulate and remain on the pen floor for extended periods of time. Depending on the size of a feedlot, it is common in Southern Alberta for pen floors to be cleaned one to two times per year followed by direct application to agricultural land [13]. While strict rules apply to manure management in order to safeguard water supplies, bacteria from fecal material can be transferred in runoff water [14]. Thus, it is valuable to understand how current agricultural practices affect dissemination of antibiotic resistance determinants into the environment. We used PCR-based methods to analyze resistance in the feces so as to include uncultured bacteria, which have been estimated to account for between 60-70% of the fecal population [15,16].

Interestingly in all fecal deposits, the concentrations of *16S-rRNA *increased in the first 56 days. Although the copy number of *16S-rRNA *per bacterial genome can vary between species [17], its quantification has previously been used to estimate overall bacterial abundance [18] and to normalize resistance genes to the bacterial population [11] in environmental samples. Our results suggest the total bacterial load in the fecal deposits increased and that the feces provided a matrix suitable for bacterial growth. This is consistent with previous reports which have identified growth of gram positive and gram negative bacteria in fecal deposits, including *E. coli *[12] and Enterococci [19].

Despite growth, not all bacteria would have proliferated. For example, as oxygen penetrated the feces, bacteria such as obligate anaerobes would have declined [20]. Temporal changes in population dynamics were reflected by DGGE patterns (Figure 6). For feces from animals that were administered antibiotics (A44, AS700, T11), DGGE patterns grouped into three main clusters that generally corresponded to early (d 7) mid (days 28 and 56) or late (days 98, 112 and 175) times of field exposure. This pattern suggests the time of exposure had a greater effect on bacterial ecology of the fecal deposits than did the type of antimicrobial fed to cattle. A notable exception to this trend was observed for DGGE patterns from control fecal deposits. Control DGGE profiles at each sampling point grouped within a single cluster that coincided with the profiles from antimicrobial-treatments on days 98, 112, and 175. As expected, the presence of tetracycline [21], tylosin [22] or sulfonamides [23] have been shown to alter bacterial populations in environment and the mammalian digestive tract. Despite being different classes of antibiotics, their effects altered the microflora of the bovine digestive tract such that bacterial populations amongst these treatments were more closely related to each other than those of control animals, an effect observed up to 98 days after being shed in feces. Bacterial populations appeared to converge in all treatments by day 98.

The community DNA used in this study originated from both live and dead bacteria however the abundance of resistance genes is an important indicator of the reservoir of antimicrobial resistance [24]. Target resistance genes were quantifiable up to day 175, indicating that bovine feces serves as a reservoir of resistance determinants for extended periods of time. The resistance determinants *tet*(L), *tet*(W), *erm*(F), and *erm*(T) genes did not increase in fecal deposits from any of the treatments and generally declined over time. In contrast, the remaining determinants in feces increased or tended to increase in concentration compared to the initial levels on day 7, followed by a decline over the remainder of the experiment. Thus the concentration of resistance genes in feces shortly after release into the environment may underestimate those at later time points. With a couple exceptions (*i.e*., *erm*(T), *erm*(X)), the overall trends of gene persistence were similar between treatments. Our data suggests that in most instances, rather than bacteria gaining or losing resistance, it was more likely that certain populations encoding resistance determinants entered a growth or death phase, respectively.

Subtherapeutic concentrations of antimicrobials have been shown to select for resistant bacteria in cattle [25,26]. Up to 75% of ingested antimicrobials have been estimated to be excreted in fecal and urine waste of livestock [27]. In the present study, the similarities in persistence of resistance genes in feces from animals fed antimicrobials to those of the control group implies that the excreted residual antimicrobials had limited selective effect on resistant bacterial populations. A previous study also found that levels of *tet*(W) and *tet*(O) did not correlate with a decrease in chlortetracycline in manure [24]. The half-lives of tetracyclines (100 days), sulfonamides (=8-30 days), and macrolides (=2-21 days) in manure are all less than the time of exposure in our study [27]. These data highlight that the selective pressure of the antimicrobials on bacteria were greater in the digestive tracts of cattle than in deposited feces. Although bovine feces has been documented as a matrix enabling the transfer of resistance genes between bacteria [28], the residual antibiotics in the feces from our study did not appear to alter gene transfer in a manner that increased overall resistance.

Tetracycline resistance genes were present in feces from all cattle, regardless of treatment. This supports previous research showing that resistance to tetracycline is widespread [29] and prevalent in the ruminant intestinal microflora even when animals are not fed antibiotics [25]. The level of resistance genes however was differentially affected by antimicrobial treatment. *tet*(B) in feces from A44 and AS700 were greater than control and T11 treatments, suggesting that chlortetracycline in the diets of animals selected for this determinant. In contrast, the concentration of *tet*(C) was greatest in deposited feces from the AS700 treatment. We have previously reported that *tet*(C) was most prevalent in ampicillin-resistant *E. coli *isolated from the feces of cattle fed AS700 as compared to A44 and control treatments [12]. The reasons for why the AS700 selects for greater levels of *tet*(C) are unknown, but may be related to the sulfamethazine in the AS700 treatment. Of the correlations between *tet*(C) and either *sul*1 or *su*l2, the strongest was observed for the AS700 treatment, providing support for this theory. Levels of *tet*(C) in feces from both A44 and T11 were greater than the control, highlighting that tylosin can also select for *tet*(C), likely through a linkage with a gene conferring resistance to macrolides. It is noteworthy however that there were only weak correlations between *tet*(C) and the *erm *genes examined in our study, perhaps indicating that linkage was with an additional gene providing resistance to tylosin.

Concentrations of *tet*(M) and *tet*(W) were clearly higher in feces as compared to the other tetracycline resistance genes. Both *tet*(M) and *tet*(W) provide resistance through ribosome protection, a mechanism of resistance generally attributed to gram positive bacteria [29]. Gram positive bacteria account for the majority of bacteria in the colon [30,31] offering an explanation as to why *tet*(M) and *tet*(W) were detected at higher levels. Previous studies have shown these determinants to be the most abundant in fecal deposits [9,10,32]. Interestingly, fecal deposits from cattle fed tylosin had higher concentrations of *tet*(W). There is evidence that some *erm *genes are linked with *tet *genes [33]. In our study, *tet*(W) had the strongest correlation to *erm*(T) and *erm*(X) in feces from cattle fed tylosin, suggesting that these determinants are linked in certain bacteria. For all fecal treatments, the concentrations of *tet*(W) declined from initial levels. A previous report found *tet*(W) to be mainly associated with obligate anaerobes [10], which may explain why there was a constant decline in this determinant in our study.

The sulfonamide resistance genes were present in higher numbers in feces from all treatments, increasing over time and in some instances being present at greater concentrations upon completion (day 175) than at initiation (day 7) of the study. Like tetracycline resistance, sulfonamide resistance is also prevalent in many *E. coli *isolated from agricultural matrices [34]. Surprisingly, levels of *sul*1 and *sul*2 were greater in A44 feces up to day 14, when compared to the other antibiotic treatments and control samples. We expected both *sul*1 and *sul*2 to be more prevalent in AS700 feces, due to the presence of sulfamethazine. Limited information exists regarding the direct effect of administering sulfonamides to cattle and development of resistance. One study showed that mixing of pig manure containing sulfadiazine with soil increased resistance in soil bacteria [23]. Additionally, *sul*1 and *sul*2 genes have been reported to increase exponentially for 60 days after storing pig manure [35], an effect similar to our results using bovine feces. Further research in this area has merit, especially considering the utility of sulfonamides in human and veterinary medicine.

In the A44 feces, the concentrations of resistance genes *erm*(A), *erm*(T) and *erm*(X) were greater compared to the control or AS700 on at least one sampling time. No obvious differences in correlations between the analyzed tetracycline resistance genes and *erm*(A), *erm*(T) and *erm*(X) existed between treatments. T11 clearly had the greatest effect on prevalence of *erm*(X), resulting in approximately a three log increase in this determinant as compared to other treatments. Chen et al.[36] reported that administering cattle tylosin resulted in greater levels of *erm*(X) in fecal grab samples compared to animals not given tylosin. Combined, these results suggest that *erm*(X) may be a useful biomarker to confirm use of tylosin in feedlots. In our study, the concentration of *erm*(X) in feces from T11-fed animals decreased from initial starting levels on day 7. This was in contrast to the concentrations of *erm*(X) in feces from cattle fed the other antibiotics or the controls, which experienced an increase in concentration followed by a decline until day 175, upon which levels were similar to those on day 7.

It is important to note that the model used in our study may have artificially introduced oxygen into the feces more rapidly than would occur in waste found in feedlot pens. The fecal deposits were contained in perforated pans and were sampled by removing feces, thus exposing random areas to ambient air. In contrast, cleaning feedlot pen floors only one to two times per year result in the accumulation of large quantities of manure at a depth that restricts oxygen concentrations. It would be expected that the microbial community and levels of resistance genes associated with anaerobes would be more stable than feces that under went a transition from anaerobic conditions in the intestinal tract to aerobic conditions on the pen floor. Our model is likely more representative of feces deposited on the pen floor as compared to that deposited in the bedding pack.

## Conclusions

Overall, this study demonstrates differential selection for resistance determinants in bovine feces depending on the type of subtherapeutic antimicrobial administered to cattle. However, the lack of consistent differences between treatment and control samples makes it difficult to predict how antimicrobials impact overall resistance. This is further compounded by the complex genetic linkages among resistance determinants. Although differences existed in the abundance of resistance genes, with the administration of antimicrobials generally selecting for higher levels of determinants, there were no statistical differences in the presence of the analyzed resistance genes in feces from cattle fed or not fed antimicrobials. We have shown that bovine feces are a long-term reservoir of resistance genes and that the density of this reservoir may increase in feces for a period of time after excretion by the animal, regardless of whether animals were administered subtherapeutic antimicrobials.

## Methods

### Animals and treatments

The study was designed so that a complete history of antimicrobial administration to the feedlot steers used for fecal collection was known and controlled, as described previously [12]. Briefly, 120 crossbred steers were randomly assigned to 12 pens. The steers received no antibiotics prior to the initiation of the experiment. Three pens (10 steers per pen) were assigned to each of four treatments: (i) control, no antibiotics; (ii) chlortetracycline (44 ppm; fed as Aureomycin-100 G; Alpharma; treatment denoted A44); (iii) chlortetracycline and sulfamethazine (each at 44 ppm; fed as Aureo S-700 G; Alpharma, Inc., Bridgewater, NJ; treatment denoted AS700); (iv) tylosin phosphate (11 ppm, fed as Tylan^®^, Elanco Animal Health; treatment denoted T11). Steers were administered antimicrobials for 197 days, starting on the day of arrival up to the point of feces collection. At the time of fecal deposit setup, steers had been fed a concentrate-based diet for the previous 96 days that consisted of 85% barley, 10% barley silage, and 5% supplement (dry matter basis). Steers assigned to the control treatment had no access to medicated feed at any time during the experiment. All cattle were cared for according to the guidelines of the Canadian Council on Animal Care [37].

### Fecal deposit preparation and sampling

For each pen, fecal samples from each steer were collected and uniformly mixed into a single composite (approx. 24 kg). The fecal material was collected in a manner that avoided feces that had contacted the ground and was added to the composite mixture within 1 min after defecation. Each composite mixture was then divided into duplicate artificial fecal deposits contained in metal pans (50 × 50 × 5 cm) to prevent possible contamination between treatments. The depth of the fecal deposits was ~ 5 cm. The bottoms of the pans were perforated to allow water to drain to the subsoil in the event of rain fall. In total, 24 fecal deposits (2 replicates per pen) were prepared. The deposits were randomly placed outside on March 1 in two adjacent rows. Ambient temperature and precipitation throughout the duration of this study are reported elsewhere [12]. Water content of fecal deposits from A44, AS700, and control animals have also been reported [12]; water content of fecal deposits from T11 animals were not different from the other treatments (data not shown).

Fecal deposits were sampled after 7, 14, 28, 42, 56, 70, 84, 98, 112, 126, and 175 days of environmental exposure. At each sampling, two subsamples (~15 g) 3 cm apart were collected and pooled. After mixing, samples were immediately taken to the lab for processing. For DNA extraction, approximately 5 g of fecal material from replicate pans were pooled together and freeze-dried (*n *= 3 per treatment). The dried material was mixed uniformly and then a subsample was ground to a powder using a planetary micro mill (Retsch, Albisheim, Germany).

### Quantification of resistance determinants

Thirty milligrams of dried fecal powder were weighed and DNA was extracted using a Qiagen QIAamp DNA Stool Mini Kit (Qiagen Inc.) according to the manufacturer's instructions, with the following exceptions: bacteria were lysed at 95°C for 10 min and 120 μl of Buffer AE were used to elute DNA from the column. DNA was quantified fluorometrically using the Quant-iT™ PicoGreen^® ^dsDNA Assay Kit (Invitrogen, Mississauga, ON) with a VersaFluor fluorometer (BioRad).

Initially, DNA from d-7 and d-56 fecal deposits of each treatment were screened by conventional PCR to detect the presence of genes encoding erythromycin (*erm*(A), *erm*(B), *erm*(F), *erm*(T), *erm*(X)), sulfonamide (*sul1, sul2*) and tetracycline (*tet*(B), *tet*(C), *tet*(L), *tet*(M), *tet*(W)) resistance, as well as *16S-rRNA*. Primers and annealing temperatures were previously described for *tet*(C), *tet*(L),and *tet*(M) [38], *tet*(B) and *tet*(W) [9], *sul*1 and *sul*2 [39], *erm*(A), *erm*(B), *erm*(F), *erm*(T), *erm*(X) [11], and *16S-rRNA *[40]. PCR for the detection of each gene was conducted individually. In addition to DNA template (20 ng), each PCR mixture (20 μl) contained (final concentrations): 1 × HotStarTaq Plus Master Mix (Qiagen Inc., Mississauga, ON) and 0.4 μM of each primer and 0.1 μg μl^-1 ^BSA (New England Biolabs), with the exception of the *tet*(C) assay, for which BSA was eliminated. The PCR conditions were: 95°C for 5 min; 35 cycles of 95°C for 20 s, respective annealing temperatures for 30 sec, 72°C for 1 min; 72°C for 10 min. The PCR were performed with an Eppendorf MasterCycler (Eppendorf, Mississauga, ON). Twenty microliters of product was visualized on a 1.5% (w/v) agarose gel, following electrophoresis and staining with ethidium bromide.

Each of the preliminary DNA samples tested positive for the genes analyzed and therefore all extracted DNA were subjected to quantitative real-time PCR. Standards for real-time PCR were created in the following way: amplicons derived from conventional PCR of the DNA samples described above, were purified using a QIAquick PCR purification kit (Qiagen Inc.) and eluted in water (pH 8) from the columns. Amplicons were then cloned into p-Drive plasmids using a Qiagen PCR Cloning Kit and transformed into Qiagen EZ competent cells, according to the manufacturer's instructions (Qiagen Inc.). The p-Drive plasmids were extracted from transformed cells using a QIAprep Spin Miniprep Kit (Qiagen Inc.), according to the manufacturer's instructions and quantified fluorometrically. Based on the p-Drive plasmid (3.85 kbp) plus amplicon size (variable), the concentration of plasmid copy numbers were calculated and diluted in 1 × TE for use in quantitative real-time PCR. To ensure the standards encoded appropriate resistant gene segments, each plasmid insert was commercially sequenced (Macrogen, South Korea) and the sequence analyzed by the BLAST feature of PubMed Nucleotide data base.

Absolute quantitative real-time PCR was performed to analyze total DNA extracted from fecal deposits. For real-time PCR, a Mastercycler ep Realplex (Eppendorf) was used. The conditions were: 95°C for 3 min; 40 cycles of 95°C for 30 sec, respective annealing temperatures for 30 sec, 72°C for 1 min. Each PCR (25 μL) contained (final concentrations): 1 × iQ SYBR Green Supermix (Bio-Rad Laboratories), 0.4 μM each primer, and 0.1 μg μl^-1 ^BSA (New England Biolabs, Pickering, ON). For *tet*(C) PCR, BSA was omitted from the reaction because of background contamination in the BSA. To each PCR, 20 ng of DNA was added. For quantification of resistant gene copy numbers, standards were prepared for each gene using the respective p-Drive plasmid containing inserted amplicons and concentrations of 10^6^, 10^5^, 10^4^, 10^3^, and 10^2 ^copies per reaction (in duplicate). Melt curve analyses were preformed on all PCR reactions to ensure specific amplification. The temperature range was 60°C to 95°C and fluorescence was measured at 0.2°C intervals.

### DGGE

DNA (200 ng) from replicate (n = 3) fecal deposits on days 7, 28, 56, 98, 112, and 175 were combined and used for PCR-DGGE analysis. The V6-V8 region of *16S-rRNA *was amplified using primers and PCR conditions described previously [41]. Amplified PCR-fragments were quantified fluorometrically as described above and 400 ng were loaded onto a polyacrylamide gel for electrophoresis using a D-Code system (Bio-Rad Laboratories) according to Huws et al.[41], with the following modifications: 6% polyacrylamide with a 40-65% gradient and electrophoresis for 20 h at 55°C, 40 V. To normalize gels for statistical analysis, a standard was made containing pooled DNA from all treated and control samples on days 7 and 175 and run every six lanes resulting in two standards per gel.

### Statistical Analysis

Gene copy numbers were log-transformed prior to statistical analysis. The persistence of genes over time was analyzed using the Mixed procedure of SAS [42]. Pen was considered the experimental unit. The model included the fixed effects of treatment (A44, AS700, T11, control), time (day of sampling), and the interaction between treatment and time. The repeated statement was applied to the day of sampling, using the pen nested within treatment as the subject. Various error structures were tested, and the one giving the lowest Akaike information criterion was chosen for analysis. Pearson correlations between different genes were analyzed using Corr procedure of SAS.

DGGE patterns of *16S rRNA *were entered into a database using the Bionumerics software (Bionumerics 5.1, Applied Maths BVBA, Sim-Martens-Latem Belgium). The patterns were analyzed using Dice similarity coefficients using unweighted pair groups methods with arithmetic average algorithms built into Bionumerics. The position tolerance and optimization was set at 1% and 0.5% respectively.

## List of Abbreviations

A44: chlortetracycline included in the diet at 44 ppm; AR: antimicrobial-resistant; AS700: chlortetracycline and sulfamethazine included in the diet each at 44 ppm; DGGE: denaturing gradient gel electrophoresis; PCR: polymerase chain reaction; T11: tylosin included in the diet at 11 ppm.

## Authors' contributions

TWA participated in study design and coordination, data analysis and drafted the manuscript. LJY participated in study design and sample collection. TR consulted on PCR analysis. RRR provided information on the relevance of the findings to human health. ET consulted on environmental implications of transmission of resistance genes. LBS assisted with study coordination. TAM was the overall project leader and participated in design and coordination of project and contributed to the final copy of the manuscript. All authors have read and approve the final manuscript.
